# Supplementary material for: The epidemiology of autoimmune bullous diseases in Sudan between 2000 and 2016
Source: PLoS One. 2021 Jul 13;16(7):e0254634. doi: 10.1371/journal.pone.0254634 (PMC8277047; doi:10.1371/journal.pone.0254634)
Supplement: S1 File — (PDF) [file pone.0254634.s001.pdf]

S1 File: the data extraction tool.

The epidemiology of autoimmune bullous diseases in Sudan between 2000 and 2016

Data Collection Sheet

Serial no.: …………….. File number: ………………………………………

Gender: 1 M 2 F Age (years): ……………..

Diagnosis: 1 PV 2 PF 3 PNP 4 IAP

5 BP 6 MMP 7 PG 8 LAD/CBDC

9 LPP 10 BSLE 11 EBA 12 DH

13 AIBD-no final diagnosis

Frequency: 1 First attack 2 Recurrent

Onset of the 1st attack (months): …………………………….

Duration of the current lesions (weeks): …………………………

Other disease? 1 No 2 Yes what?..
