# Supplementary material for: The epidemiology of autoimmune bullous diseases in Sudan between 2000 and 2016
Source: PLoS One. 2021 Jul 13;16(7):e0254634. doi: 10.1371/journal.pone.0254634 (PMC8277047; doi:10.1371/journal.pone.0254634)
Supplement: S1 Table — (PDF) [file pone.0254634.s002.pdf]

S2 Table: the findings of the studies that investigated the whole spectrum of AIBDs including our study.

	Present Study (2001-2016)	Iran (1997-2006)	Tunisia (1997-2007)	Romania (April 2001-Dec. 2007)	Germany (Jan. 2001-June 2002)	Kuwait (July 1991- Dec. 2002)	China (1953-1991)	Malaysia (15 years)
**Intraepidermal group**	N = 355(60.7%)[a]<br>Age[b] = 41.1<br>M:F = 1:2.3	N = 1207 (86.1%)<br>Age = 43.3<br>M:F = 1:1.4	N = 92 (52.9%)<br>Age = 50.0<br>M:F = 1:2.1	N = 68 (58.6%)<br>Age = NR<br>M:F = NR	N = 1 (2.4%)<br>Age = 62<br>M:F = NR	N = 60 (46.9%)<br>Age = 36.5<br>M:F = 1:0.9	N = 827 (84.1%)<br>Age = mostly 40- 60<br>M:F = 1:0.8	N = 84(56.7%)<br>Age = mostly 30-60<br>M:F = 1:1
**PV**	N = 298 (50.9%)<br>Age = 40.4<br>M:F = 1:2.3	N = 1138 (81.2%)<br>Age = 43.4<br>M:F = 1:1.4	N = 56 (32.2%)<br>Age = NR<br>M:F = 1:2.1	N = 55 (47.4%)<br>Age = 53<br>M:F = 1:1.8	N = 1 (2.4%)<br>Age = 62<br>M:F = NR	N = 48 (37.5%)<br>Age = 36.5<br>M:F = 1:0.7	N = 372 (37.8%)<br>Age = NR<br>M:F = NR	N = 84(56.7%)<br>Age = mostly 30-60<br>M:F = 1:1
**PF**	N = 48 (8.2%)<br>Age = 46.3<br>M:F = 1:2.2	N = 63 (4.5%)<br>Age =42.2<br>M:F = 1:1.3	N = 34 (19.5%)<br>Age = NR<br>M:F = 1:1.8	N = 9 (7.8%)<br>Age = NR<br>M:F = NR	N = 0<br>Age = NA<br>M:F = NA	N = 11 (8.6%)<br>Age = 36.9<br>M:F = 1:2.7	N = 448 (45.6%)<br>Age = NR<br>M:F = NR	N = 0<br>Age = NA<br>M:F = NA
**PNPP**	N = 2 (0.3%)<br>Age = 51.5<br>M:F = 2:0	N = 3 (0.2%)<br>Age = 35.7<br>M:F = 1:2	N = 1 (0.6%)<br>Age = NR<br>M:F = NR	N = 2 (1.7%)<br>Age = NR<br>M:F = NR	N = 0<br>Age = NA<br>M:F = NA	NI	N = 5[c] (0.5%)<br>Age = NR<br>M:F = NR	N = 0<br>Age = NA<br>M:F = NA
**IAP**	N = 7 (1.2%)<br>Age = 33.3<br>M:F = 1:6	N = 3 (0.2%)<br>Age = 27.7<br>M:F = 1:0.5	N = 1 (0.6%)<br>Age = NR<br>M:F = NR	N = 1 (0.9%)<br>Age = NR<br>M:F = NR	N = 0<br>Age = NA<br>M:F = NA	N = 1 (0.8%)<br>Age = 35<br>M:F = 0:1	N = 2 (0.2%)<br>Age = NR<br>M:F = NR	N = 0<br>Age = NA<br>M:F = NA

**Subepidermal Group**	N = 230 (39.3%)<br>Age = 51.6<br>M:F = 1:1	N = 195 (13.9%)<br>Age = 55.3<br>M:F = 1:1.4	N = 82 (47.1%)<br>Age = NR<br>M:F = NR	N = 48 (41.4%)<br>Age = NR<br>M:F = NR	N = 40 (97.6%)<br>Age = NR<br>M:F = NR	N = 68 (53.1%)<br>Age = NR<br>M:F = NR	N = 156 (15.9%)<br>Age = 1- 84<br>M:F = 1:0.8	N = 64(43.2%)<br>Age = NR<br>M:F = 1:1.3
**BP**	N = 165 (28.2%)<br>Age = 66.0<br>M:F = 1:0.9	N = 163 (11.6%)<br>Age = 59.4<br>M:F = 1:1.4	N = 41 (23.6%)<br>Age = 68.6<br>M:F = 1:0.6	N = 40 (34.5%)<br>Age = 73<br>M:F = 1:1.5	N = 27 (65.9%)<br>Age = 64.6<br>M:F = 1:1.2	N = 27 (21.1%)<br>Age = 66<br>M:F = 1:5.8	N = 104 (10.6%)<br>Age = 10- 84<br>M:F = 1:0.7	N = 51(34.5%)<br>Age = mostly 40-80<br>M:F = 1:1.2
**MMP**	N = 1 (0.2%)<br>Age = 6.0<br>M:F = 0:1	N = 10 (0.7%)<br>Age = 45.5<br>M:F = 1:1.2	N = 2 (1.1%)<br>Age = 73.5<br>M:F = 2:0	N = 0<br>Age = NA<br>M:F = NA	N = 4 (9.8%)<br>Age = 76.3<br>M:F = NR	N = 1 (0.8%)<br>Age = 50<br>M:F = 1:0	N = 9 (0.9%)<br>Age = 14- 52<br>M:F = 1:1.3	N = 0<br>Age = NA<br>M:F = NA
**PG**	N = 8 (1.4%)<br>Age = 29.5	N = 10 (0.7%)<br>Age = 27.7	N = 18 (10.3%)<br>Age = 31.7	N = 1 (0.9%)<br>Age = NR	N = 4 (9.8%)<br>Age = 33	N = 24 (18.8%)<br>Age = 28.6	N = 0<br>Age = NA	N = 0<br>Age = NA
**LAD/ CBDC**	N = 49 (8.4%)<br>Age = 9.0<br>M:F = 1:1	N = 5 (0.4%)<br>Age = 22.4<br>M:F = 1:0.7	N = 11 (6.3%)<br>Age = 18<br>M:F = 1:1.6	N = 3 (2.6%)<br>Age = NR<br>M:F = NR	N = 2 (4.9%)<br>Age = 31<br>M:F = NR	N = 9 (7.0%)<br>Age = 12.7<br>M:F = 1:0.6	N = 36 (3.7%)<br>Age = 1- 78<br>M:F = 1:1	N = 13[d](8.8%)<br>Age = mostly< 30<br>M:F = 1:1.6
**LPP**	N = 1 (0.2%)<br>Age = 16.0<br>M:F = 0:1	N = 0<br>Age = NA<br>M:F = NA	NI	N = 1 (0.9%)<br>Age = NR<br>M:F = NR	N = 0<br>Age = NA<br>M:F = NA	N = 4 (3.1%)<br>Age = 42.8<br>M:F = 0:4	N = 0<br>Age = NA<br>M:F = NA	N = 0<br>Age = NA<br>M:F = NA
**BSLE**	N = 1 (0.2%)<br>Age = 38.0<br>M:F = 0:1	N = 0<br>Age = NA<br>M:F = NA	NI	N = 1 (0.9%)<br>Age = NR<br>M:F = NR	N = 0<br>Age = NA<br>M:F = NA	N = 0<br>Age = NA<br>M:F = NA	N = 0<br>Age = NA<br>M:F = NA	N = 0<br>Age = NA<br>M:F = NA

**EBA**	N = 0<br>Age = NA<br>M:F = NA	N = 7 (0.5%)<br>Age = 40.3<br>M:F = 1:0.2	N = 1 (0.6%)<br>Age = 53<br>M:F = 0:1	N = 2 (1.7%)<br>Age = NR<br>M:F = NR	N = 1 (2.4%)<br>Age = 36<br>M:F = NR	N = 3 (2.3%)<br>Age = 59.1<br>M:F = 0:3	N = 7 (0.7%)<br>Age = 2- 67<br>M:F = 1:0.8	N = 0<br>Age = NA<br>M:F = NA
**DH**	N = 5 (0.9%)<br>Age = 49.2<br>M:F = 1:0.3	NI	N = 9 (5.2%)<br>Age = 26<br>M:F = 1:2	N = 0<br>Age = NA<br>M:F = NA	N = 2 (4.9%)<br>Age = 42.5<br>M:F = NR	NI	N = 0<br>Age = NA<br>M:F = NA	N = 0<br>Age = NA<br>M:F = NA
**Total**	N = 585<br>Age = 45.2<br>M:F = 1:1.6	N = 1402<br>Age = 45<br>M:F = 1:1.4	N = 174<br>Age = 0.8 - 102<br>M:F = 1:1.7	N = 116<br>Age = NR<br>M:F = NR	N = 41<br>Age = NR<br>M:F = NR	N = 128<br>Age = 42<br>M:F = 1:2.1	N = 983<br>Age = NR<br>M:F = NR	N = 148<br>Age = NR<br>M:F = 1:1.1
**Duration[e]**	16	10	11	6.75	1.5	10.5	38	15
**Rate[f]**	57.7	140.2	15.8	17.2	27.3	11.1	11.8	9.9

[a]Percentage represents the relative frequency.

[b]Age (in years) is represented by the mean age at presentation if available, otherwise the age range is used instead.

[c]In the original paper, it was reported as pemphigus associated malignancy.

[d]In the original paper, it was reported as dermatitis herpetiformis with linear IgA deposition.

[e]Period covered by the study in years.

[f]Number of cases per year.

BP, bullous pemphigoid; BSLE, bullous systemic lupus erythematosus; DH, dermatitis herpetiformis; EBA, epidermolysis bullosa acquisita; IAP, immunoglobulin A pemphigus; LAD/ CBDC, linear immunoglobulin A disease/ chronic bullous disease of the childhood; LPP, lichen planus pemphigoides; m:f , male to female ratio; MMP, mucous membrane pemphigoid; n, number of cases; NA, not applicable; NI, not included in the study; NR, not reported; PF, pemphigus foliaceous; PG, pemphigoid gestationis; PNPP, paraneoplastic pemphigus; PV, pemphigus vulgaris.
