# Supplementary material for: The epidemiology of autoimmune bullous diseases in Sudan between 2000 and 2016
Source: PLoS One. 2021 Jul 13;16(7):e0254634. doi: 10.1371/journal.pone.0254634 (PMC8277047; doi:10.1371/journal.pone.0254634)
Supplement: Attachment — Submitted filename: AIBDs - Rebuttal letter.docx [file pone.0254634.s003.docx]

Omayma Siddig

Faculty of Dentistry, University of Khartoum

Khartoum 11111

Sudan

20^th^ of January, 2021

Ref.: PONE-D-20-35931, The epidemiology of autoimmune bullous diseases in Sudan between 2000 and 2016, PLOS ONE

Dear Amit Sapra

We thank you and the reviewer for the generous comments on the manuscript that have definitely helped improve the manuscript.

We have edited the manuscript to address the raised points as follows:

Journal requirements:

1) We double checked that the manuscript meets PLOS ONE's style requirements.
2) We included the data extraction tool as Supporting Information, named S1_File. We re-uploaded the previous S1_File.pdf as S2_File. We developed the tool in English and therefore we are uploading it as it is.
3) Standard reporting of the rates was used (lines 25, 74).
4) Regarding data availability, the ethics committees of the University of Khartoum and Khartoum State Ministry of Health restricted publicly sharing of research data that were extracted from routinely-collected healthcare data even if anonymized. Some of the diseases under study are very rare and therefore potentially identifying. Data are available from the University of Khartoum Faculty of Dentistry ([dentistry@uofk.edu](mailto:dentistry@uofk.edu)).

Reviewer’s comments:

1) The study design is now written as advised (lines 18 and 57).
2) We agree that a lack of consent is an ethical consideration in general. However, for a retrospective study using routinely-collected health care data, this is a grey area. The ethics committees exempted this study from the requirement of informed consent based on its minimal risk to ethics.
3) Grammatical errors were corrected in lines: 19, 24, 35, 50,64, 65, 78, 123, 132, 188, 212, and 240.
4) We added information on gender percentages for each disease to Table 1. We still believe that the male-to female ratio is important for comparison with other countries.
5) A bar chart for age representation is uploaded as Fig4 and appropriately cited within the manuscript.
6) We did not observe a high prevalence of a co-morbidity with a specific AIBDs.

Attached along with this letter are the marked-up manuscript with track changes, the unmarked manuscript, the added figure and the supplementary files.

We look forward to receiving from you the acceptance of publication in PLOS ONE. We would be happy to respond to any questions or comments that may arise.

Kind regards,

Omayma Siddig; BDS, MFD, MFDS, MSc

Faculty of Dentistry, University of Khartoum

On behalf of all authors.
