# Supplementary material for: The epidemiology of autoimmune bullous diseases in Sudan between 2000 and 2016
Source: PLoS One. 2021 Jul 13;16(7):e0254634. doi: 10.1371/journal.pone.0254634 (PMC8277047; doi:10.1371/journal.pone.0254634)
Supplement: Attachment — Submitted filename: AIBDs - Rebuttal letter.docx [file pone.0254634.s004.docx]

Omayma Siddig

Faculty of Dentistry, University of Khartoum

Khartoum 11111

Sudan

29 June, 2021

Ref.: PONE-D-20-35931, The epidemiology of autoimmune bullous diseases in Sudan between 2000 and 2016, second revision

Dear Feroze Kaliyadan

We thank you for the flexibility you showed regarding the due date for submission this manuscript. We also thank the reviewer for their time reviewing and commenting on the manuscript.

We have edited the manuscript as requested and here are the details of the changes made:

Journal requirements:

1) Regarding reference list: we removed the reference related to the reviewer’s first comment, and we added a reference reporting on the outcomes of COVID-19 in patients with autoimmune bullous as we believe it enforces the idea of a national registry.
2) We uploaded the pictures that have been corrected by the PACE tool as advised.

Reviewer’s comments:

1) We deleted the phrase “suggesting an element of gender inequality in those countries”. The reviewer made a valid point and we agree with them.
2) Although the association between neurological diseases and autoimmune bullous diseases is an interesting area and yes our study lacked any information about neurological diseases, we cannot comment on this issue. We feel that it would be biased to comment on association between neurological diseases and autoimmune bullous diseases only and leave all other associations that have been reported in the literature as if we imply that there is definitely an association. On the other hand, we cannot comment on every single association that was reported as it would be overwhelming for the reader and would put the manuscript off track.

Attached along with this letter are the marked-up manuscript with track changes, the unmarked manuscript, the PACE-corrected figures and the supplementary files.

We look forward to receiving from you the acceptance of publication in PLOS ONE. We would be happy to respond to any questions or comments that may arise.

Kind regards,

Omayma Siddig; BDS, MFD, MFDS, MSc

Faculty of Dentistry, University of Khartoum

On behalf of all authors.
